# IQGAP1: cross-disease target via receptor-pathway networks

**DOI:** 10.3389/fonc.2025.1632060

**Published:** 2025-09-16

**Authors:** Shaopeng Zhu, Yunpeng Zou, Jie Guo, Wenqi Ma, Laitong Lu, Ronghan Liu, Jianning Kang, Kai Zhao, Jiangbo Zhong

**Affiliations:** ^1^ School of Clinical Medicine, Shandong Second Medical University, Weifang, Shandong, China; ^2^ Central Hospital Affiliated to Shandong First Medical University, Shandong First Medical University and Shandong Academy of Medical Sciences, Jinan, Shandong, China; ^3^ Department of Spin Surgery, Central Hospital Affiliated to Shandong First Medical University, Jinan, Shandong, China

**Keywords:** IQGAP1, scaffold protein, cancer, drug resistance, immune modulation, metabolic diseases, cell signaling, therapeutic target

## Abstract

IQGAP1, a versatile scaffolding protein, critically regulates cytoskeletal organization, cell motility, proliferation, and signaling cascades. Beyond coordinating these cellular functions, it is increasingly recognized as a key driver in malignancies, immune dysfunction, metabolic dysregulation, and cardiovascular pathologies. By binding receptor tyrosine kinases, small GTPases, and downstream effectors, IQGAP1 modulates oncogenesis, immune evasion, and metabolic imbalance, while contributing to chemoresistance. This review synthesizes advances in IQGAP1’s structural domains, disease-specific signaling networks, and therapeutic targeting strategies, emphasizing its translational potential in developing precision therapies for cancer, metabolic syndromes, and immune disorders.

## 1 Introduction

IQ motif containing GTPase-activating protein 1 (IQGAP1) is a multifunctional scaffold protein that plays an essential role in regulating various biological processes, including cytoskeletal dynamics, cell migration, proliferation, signal transduction, and cell-cell adhesion. Since its initial discovery, IQGAP1 has been extensively studied for its pivotal role in controlling cell morphology and motility. This protein exerts its effects through interactions with numerous signaling molecules and receptors, regulating fundamental cellular functions and influencing a variety of physiological and pathological states. The significance of IQGAP1 has expanded beyond its basic cellular roles, revealing its critical involvement in various diseases, particularly cancer, immune disorders, and metabolic diseases.

As a member of the IQGAP protein family, which also includes IQGAP2 and IQGAP3, IQGAP1 was first identified in human osteosarcoma cells. IQGAP1 is a large molecular entity, with a molecular weight of approximately 190 kDa, composed of 1657 amino acids. It is widely expressed in human tissues and possesses five major functional domains: a calmodulin homology domain (CHD), a proline-rich WW domain involved in protein-protein interactions, an IQ domain, a Ras GAP-related domain (GRD), and a C-terminal Ras GAP domain (RGCT) (1, 2). These structural features enable IQGAP1 to interact with key signaling pathways, thus modulating critical cellular processes.

IQGAP1 has emerged as a crucial regulator in many physiological contexts. For instance, in the kidney, IQGAP1 is involved in the regulation of the glomerular filtration barrier by co-localizing with renin during the foot process of podocytes, affecting the permeability of the glomerular basement membrane (3, 4). In the nervous system, IQGAP1 plays a critical role in neuronal proliferation and migration, which is vital for proper neuronal network formation. Notably, in the absence of IQGAP1, vascular endothelial growth factor (VEGF) fails to stimulate the migration and differentiation of neural progenitors into neurons, highlighting its importance in neurogenesis (5, 6). Additionally, in the cardiovascular system, IQGAP1 has been shown to be indispensable for adaptive remodeling in response to chronic hemodynamic stress, such as in heart failure, where its absence leads to maladaptive cardiac remodeling, ventricular wall thinning, and reduced contractile function (7).

The role of IQGAP1 in cancer has garnered significant attention. It is implicated not only in tumor cell proliferation, migration, and metastasis but also in chemoresistance, making it a potential target for therapeutic intervention (8). IQGAP1 mediates the activation of multiple signaling pathways, including *EGFR* and *CXCR4*, which are crucial for tumor cell behavior. In particular, its interaction with EGFR enhances receptor activation, thereby promoting tumor progression (2). Furthermore, in immune regulation, IQGAP1 has been identified as a key modulator of T-cell activation and immune evasion, with significant implications for immune-based therapies (8, 9).

Beyond cancer and immunity, IQGAP1 has shown relevance in metabolic disorders. Studies suggest that IQGAP1 plays a vital role in regulating metabolic homeostasis, fat cell function, and insulin sensitivity, with potential implications for diseases like obesity and diabetes (10, 11). Its involvement in these diverse biological processes underscores its importance as a potential therapeutic target, not only for cancer but also for a range of immune and metabolic disorders.

In summary, IQGAP1’s multifaceted roles in cellular regulation, cancer progression, immune modulation, and metabolic disease highlight its potential as a therapeutic target. As research progresses, understanding the precise mechanisms through which IQGAP1 influences these processes will be crucial in developing targeted therapies for a variety of diseases. The diverse biological functions of IQGAP1 and its ability to integrate cellular signals across different systems make it an exciting candidate for future therapeutic strategies.

## 2 Structure and function of IQGAP1

IQGAP1 is a large, multifunctional scaffold protein of approximately 190 kDa. It plays a crucial role in regulating a variety of cellular processes, including cytoskeletal dynamics, cell signaling, motility, and proliferation. Unlike classic GTPase-activating proteins (GAPs), IQGAP1 does not have intrinsic GAP activity but instead serves as an integrator of multiple signaling pathways through its interaction with various binding partners (8, 12).

### 2.1 Structural domains of IQGAP1

IQGAP1 consists of several structural domains that mediate its multifunctional roles in the cell. These include:

Calponin Homology Domain (CHD): This domain is responsible for binding to actin filaments, which allows IQGAP1 to play a central role in regulating the cytoskeleton and cellular shape (13).

WW Domain: A proline-rich motif that enables interaction with proteins containing SH3 domains. This domain is crucial for mediating protein-protein interactions in various signaling pathways (13).

IQ Domain: Containing four IQ motifs, this domain binds to calmodulin, allowing IQGAP1 to mediate calcium-dependent and -independent signaling processes. The IQ domain plays a role in integrating signals from the intracellular environment (8).

RasGAP-Related Domain (GRD): This domain interacts with small GTPases such as Cdc42 and Rac1. These GTPases regulate a variety of cellular functions, including cell polarity, migration, and adhesion. IQGAP1 helps modulate the activity of these GTPases, linking them to downstream signaling events (13).

**Figure 1:**
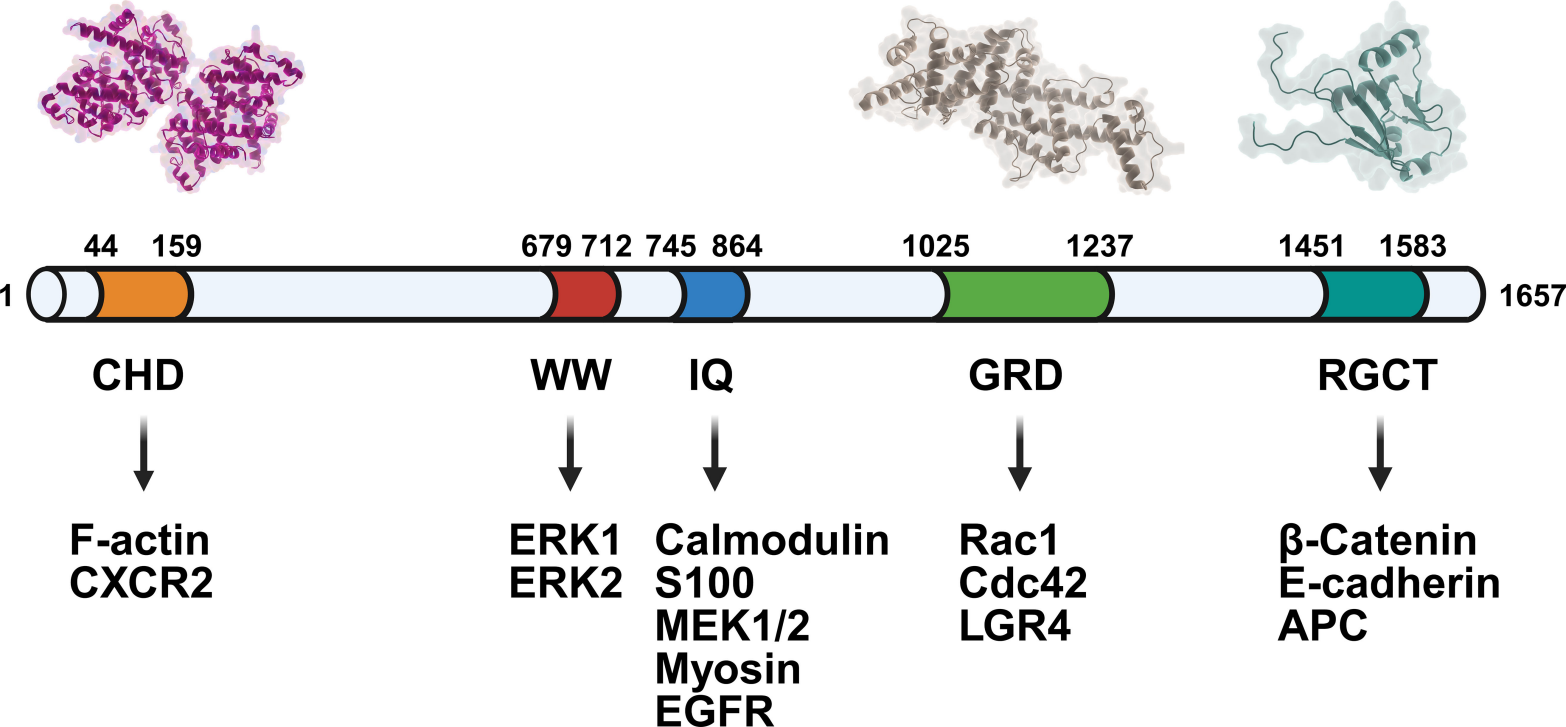
Domain architecture of IQGAP1 and its interaction partners. The schematic diagram illustrates the modular structure of IQGAP1, highlighting key functional domains and their associated binding partners. From the N-terminus to the C-terminus: the CHD domain interacts with F-actin and CXCR2; the WW domain binds to ERK1 and ERK2; the IQ domain binds to various proteins, including calmodulin, S100, MEK1/2, actin, and EGFR; the GRD domain mediates interactions with Rac1, Cdc42, and LGR4; and the RGCT domain participates in binding to β-catenin, E-cadherin, and APC.

RasGAP C-Terminal Domain (RGCT): This domain is involved in mediating protein-protein interactions, contributing to IQGAP1’s function as a scaffold that assembles signaling complexes. The RGCT domain plays a key role in regulating cellular responses to external stimuli (14) (**Figure 1**).

### 2.2 Functional roles of IQGAP1

IQGAP1 plays a critical role in regulating cellular processes by acting as a scaffold protein that integrates and coordinates various signaling pathways. It interacts with multiple signaling molecules, including receptor tyrosine kinases (e.g., EGFR, HER2, FGFR1), small GTPases (e.g., Rac1, Cdc42), and downstream effectors (e.g., *B-Raf, MEK, ERK, PI3K/Akt*). Through these interactions, IQGAP1 modulates processes such as cell proliferation, migration, and differentiation. It is involved in key signaling pathways, including *MAPK, PI3K/Akt, Wnt/β-catenin, and Hippo*, all of which are essential for normal cell function and tissue homeostasis.

In addition, IQGAP1 regulates the cytoskeleton by modulating actin dynamics and cell motility, which is important for processes like wound healing, immune response, and cancer metastasis. IQGAP1’s activity is further regulated by post-translational modifications, such as SUMOylation, which enhances its stability and signaling functions. IQGAP1’s ability to integrate multiple cellular signals makes it a crucial regulator of both normal physiology and disease progression (2, 15, 16).

## 3 Relationship between IQGAP1 and cancer

IQGAP1 is increasingly recognized as a master integrator of oncogenic signals, acting as a versatile scaffold that coordinates diverse cellular pathways fundamental to tumorigenesis, metastasis, and therapy resistance. Overexpressed in a broad spectrum of cancers, IQGAP1 facilitates tumor progression by interfacing with membrane-bound receptors and cytoplasmic signaling mediators, orchestrating a network of molecular interactions critical to cancer cell survival and dissemination.

### 3.1 Interaction of IQGAP1 with cancer receptors

#### 3.1.1 Receptor tyrosine kinases and their pathways

IQGAP1 interacts with a variety of receptor tyrosine kinases (RTKs), acting as a central regulator of signaling cascades that promote tumor progression.

##### 3.1.1.1 EGFR (epidermal growth factor receptor)

IQGAP1 binds to EGFR through its IQ domain, regulating EGFR phosphorylation and promoting downstream signaling via the *MAPK* and *PI3K/AKT* pathways. This interaction is crucial for tumor cell proliferation, migration, and survival, particularly in head and neck cancers. Phosphorylation of IQGAP1 at Ser1443 by PKCα modulates EGFR-mediated signaling, impacting PD-L1 expression (2, 17).

##### 3.1.1.2 HER2 (human epidermal growth factor receptor 2)

In breast cancer, IQGAP1 directly interacts with HER2, regulating its phosphorylation and stability. This interaction is essential for HER2-driven oncogenesis, contributing to increased cell survival and proliferation (18).

##### 3.1.1.3 MET (hepatocyte growth factor receptor)

IQGAP1 associates with MET and is phosphorylated at Tyr^1510^, facilitating the docking of SH2 domain-containing proteins such as Abl1 and Abl2. It modulates MET signaling, promoting cell migration, invasion, and metastasis. This dual regulatory role positions IQGAP1 as a key mediator of MET-driven cancers (19).

##### 3.1.1.4 FGFR1 (fibroblast growth factor receptor 1)

IQGAP1 functions as a scaffold for FGF2-induced signaling, enhancing cytoskeletal reorganization through N-WASP and Arp2/3. This process is critical for fibroblast-like cell migration during tumor progression (20).

#### 3.1.2 Chemokine receptors and tumor cell migration

IQGAP1 also plays an important role in modulating chemokine receptor signaling, which is vital for cancer cell migration and metastasis.

##### 3.1.2.1 CXCR4 (chemokine receptor 4)

IQGAP1 regulates CXCR4 receptor trafficking and recycling, promoting SDF-1-induced migration. It enhances downstream ERK activation, facilitating leukemia and carcinoma cell migration and invasion (21).

##### 3.1.2.2 CXCR2 (chemokine receptor 2)

IQGAP1 interacts with CXCR2, promoting its localization at the cell front and supporting chemotactic migration in neutrophils. This interaction also plays a crucial role in tumor cell navigation within the tumor microenvironment (22).

#### 3.1.3 Integrins and adhesion receptors

IQGAP1 influences cell adhesion and motility through its interactions with integrins and other adhesion receptors, driving cancer metastasis.

##### 3.1.3.1 β1-integrin

IQGAP1 enhances β1-integrin transcription and stability via the ERK/FAK and SRF pathways. This regulation is important for metastatic colonization, especially in lung metastases associated with breast and prostate cancers (23).

##### 3.1.3.2 CD44

IQGAP1 also interacts with CD44, the hyaluronan receptor, to promote actin cytoskeletal rearrangement and focal adhesion formation. This interaction is crucial for cancer cell migration and tumor progression, particularly in fibroblast-like cells (24).

#### 3.1.4 Downstream signaling pathways

IQGAP1 serves as a scaffold for several critical intracellular signaling pathways that regulate tumor growth, survival, and metastasis.

##### 3.1.4.1 MAPK/ERK pathway

IQGAP1 facilitates the activation of the *MAPK* cascade by binding to B-Raf, MEK, and ERK. This pathway is essential for sustained mitogenic signaling, contributing to cancer cell proliferation, survival, and response to growth factors (25).

##### 3.1.4.2 PI3K/AKT pathway

IQGAP1 interacts with PI3Kα and PDK1, promoting PIP3 synthesis and Akt activation. This signaling pathway is critical for promoting cell survival and resistance to apoptosis, particularly in response to growth factor stimulation (15).

##### 3.1.4.3 Hippo/YAP pathway

IQGAP1 interacts with YAP1, inhibiting MST2 and LATS1 kinases in the Hippo pathway. This regulation prevents cell death and promotes liver tumorigenesis, emphasizing IQGAP1’s role in promoting cancer cell survival (26).

##### 3.1.4.4 mTOR pathway

IQGAP1 modulates *mTOR signaling*, supporting Akt phosphorylation and promoting tumor progression, particularly in hepatocellular carcinoma (27).

#### 3.1.5 Small GTPases and cytoskeletal dynamics

IQGAP1 regulates small GTPases and is essential for cytoskeletal dynamics, which are crucial for cancer cell motility and metastasis.

##### 3.1.5.1 Rac1 and Cdc42

IQGAP1 acts as a scaffold for Rac1 and Cdc42, promoting actin cytoskeletal reorganization. This facilitates tumor cell migration and invasion, as seen in hepatocellular carcinoma, where IQGAP1 enhances Rac1-dependent Src/FAK signaling (28).

##### 3.1.5.2 ARF1

IQGAP1 interacts with ARF1, contributing to ERK reactivation in vemurafenib-resistant colorectal cancer. This interaction underscores IQGAP1’s role in overcoming therapeutic resistance by facilitating the reactivation of key signaling pathways (29).

### 3.2 The role of IQGAP1 in tumor drug resistance

IQGAP1 has been implicated in the regulation of tumor drug resistance across various cancer types. By modulating different molecular pathways, IQGAP1 facilitates tumor cell adaptation to chemotherapy, radiotherapy, and targeted therapies, thus contributing to the development of drug resistance.

In esophageal squamous cell carcinoma (ESCC), IQGAP1 enhances resistance to paclitaxel (PTX) by upregulating the Hippo pathway downstream effector YAP. Specifically, IQGAP1 overexpression activates YAP, which in turn inhibits ferroptosis, thereby promoting resistance to PTX. This mechanism was validated through the use of ferroptosis agonists (RSL3) and inhibitors (Fer-1), which demonstrated that IQGAP1-mediated regulation of ferroptosis plays a crucial role in the development of resistance. These findings highlight the potential of targeting the IQGAP1-YAP axis as a therapeutic strategy for overcoming PTX resistance in ESCC (30).

In hepatitis B virus (HBV)-positive hepatocellular carcinoma (HCC), IQGAP1 is upregulated by increased reactive oxygen species (ROS) levels, which promotes its binding to Rac1 and subsequently activates the *Src/FAK signaling* axis. This pathway enhances tumor cell survival and migration, thereby contributing to therapy resistance. Notably, the use of antioxidant treatments that inhibit ROS production was able to reverse the resistance phenotype, suggesting that targeting the ROS/IQGAP1 axis could be a viable strategy to sensitize tumors to treatment (28).

Additionally, in rectal adenocarcinoma, IQGAP1 plays a pivotal role in mediating resistance after radiation and chemotherapy. Post-treatment tumor samples exhibited elevated IQGAP1 expression, particularly in patients with poor treatment responses. IQGAP1 activation of the *MAPK pathway* was identified as a key mechanism driving the post-treatment resistance phenotype. These observations suggest that IQGAP1 expression levels could serve as a predictive biomarker for therapeutic response, offering potential for personalized treatment approaches (31).

Finally, IQGAP1 also contributes to resistance in targeted therapies for breast cancer. By forming a complex with ARF1, IQGAP1 activates the *ERK signaling pathway*, which enhances tumor cell invasiveness and confers resistance to BRAFV600E inhibitors, such as vemurafenib. Disruption of the IQGAP1-ARF1 interaction using specific inhibitors (e.g., LY2835219) was shown to reverse ERK activation and restore drug sensitivity, further emphasizing IQGAP1’s role in therapeutic resistance (29).

In conclusion, IQGAP1 mediates tumor drug resistance through multiple mechanisms, including the suppression of ferroptosis via YAP activation, ROS-induced *Src/FAK signaling*, sustained MAPK activation, and ARF1-IQGAP1 complex-driven ERK activation. These findings underscore the potential of IQGAP1 as a therapeutic target in overcoming drug resistance, particularly by targeting its downstream signaling pathways. Such strategies could pave the way for novel therapeutic interventions in cancer treatment.

### 3.3 IQGAP1 in cancer

#### 3.3.1 Head and neck cancer

Head and neck cancer is the sixth most common cancer globally (32). Among its causes, HPV infection is a significant factor. The oncogenic proteins of HPV16 activate the *PI3K pathway*, influencing the pathogenicity of head and neck cancer and affecting its prognosis (33–35). In a mouse papillomavirus model, IQGAP1 promotes cell migration through the highly active *PI3K/AKT/mTOR signaling pathway*. However, IQGAP1 does not affect the infectivity of the virus. Interestingly, IQGAP1 also does not influence the carcinogenicity of HPV16 transgenic mice (33).

IQGAP1 regulates the phosphorylation of splicing proteins via the PI3K pathway, thereby altering splicing activity without affecting the overall protein levels. This modulation results in cancer-related splicing events (34). Additionally, HPV oncogenic proteins activate the *Rac1/Cdc42 pathway* and mediate cancer cell invasion and migration through their interaction with IQGAP1 (36, 37).

**Figure 2:**
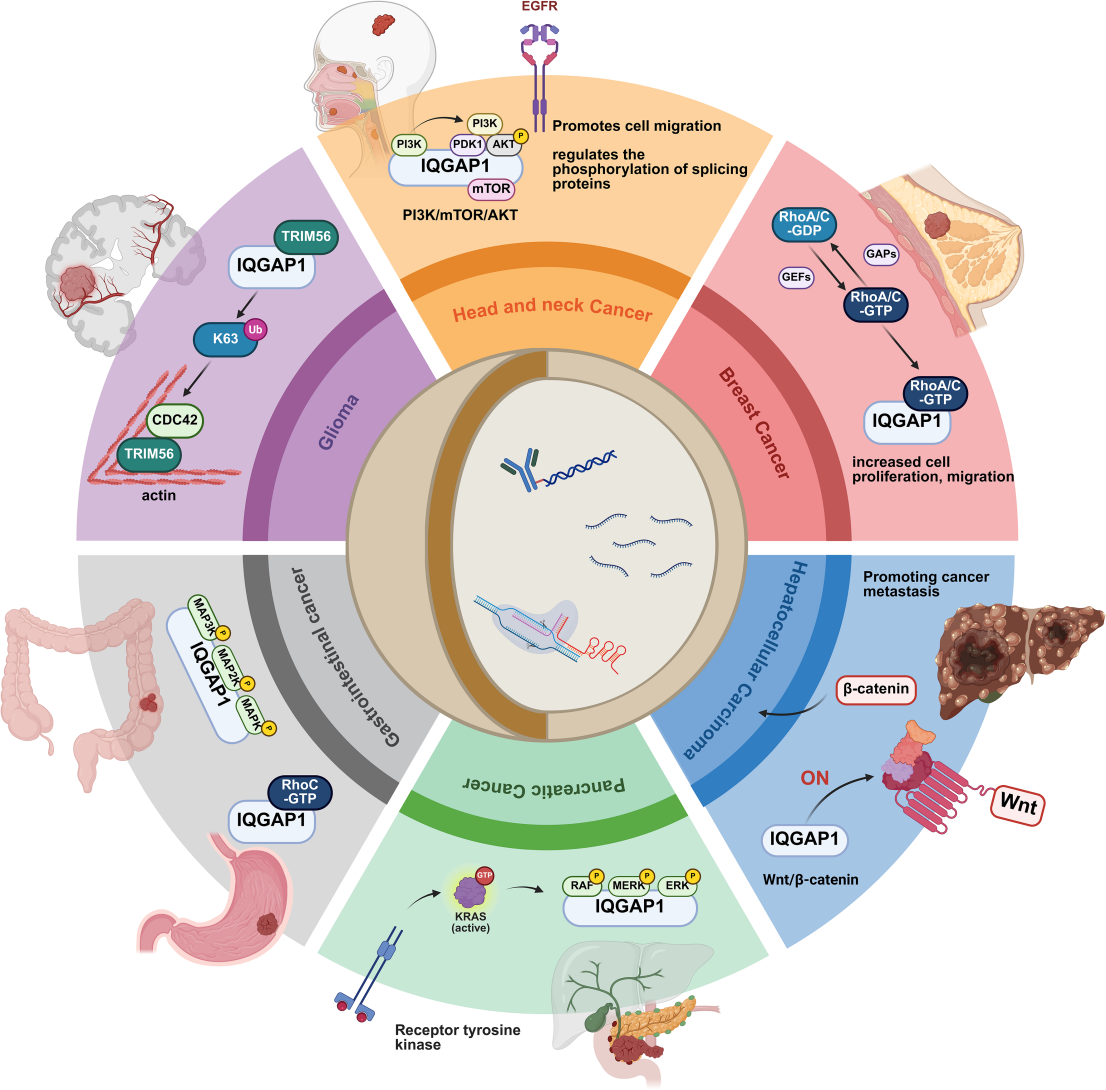
IQGAP1-mediated signaling in cancer progression. IQGAP1 plays a key role in cancer progression by regulating key signaling pathways associated with cell migration, proliferation, and metastasis. In head and neck cancers, IQGAP1 regulates EGFR signaling through the PI3K/mTOR/AKT pathway, promoting cell migration and altering the phosphorylation of splicing proteins. In breast cancer, IQGAP1 activates RhoA/C GTPases, enhancing cell proliferation and migration. In hepatocellular carcinoma, IQGAP1 promotes the Wnt/β-catenin pathway, leading to increased cell proliferation and metastasis. In pancreatic cancer, IQGAP1 regulates the KRAS signaling pathway, activating the MAPK/ERK pathway to support tumor survival and spread. In gastrointestinal cancer, IQGAP1 is associated with RhoC GTPases and the MAPK signaling pathway, driving cell growth and migration. Finally, in gliomas, IQGAP1 interacts with TRIM56 and CDC42, influencing actin dynamics and cell motility. This figure illustrates the multifaceted role of IQGAP1 in regulating key pathways across different cancer types, highlighting its central role in cancer progression.

Given the critical role of the *IQGAP1/PI3K pathway* in the development of head and neck cancer, IQGAP1 has emerged as a potential therapeutic target for controlling this type of cancer (**Figure 2**).

#### 3.3.2 Breast cancer

Breast cancer is the most common malignant tumor in women, accounting for approximately one-quarter of all cases, and it poses a serious threat to the health and lives of women worldwide (32). Based on pathological and immunohistochemical markers, breast cancer can be classified into four subtypes: Luminal A (the most common molecular subtype: ER+, PR+, HER2-), Luminal B (ER+, PR+, HER2+), HER2-positive (ER-, PR-, HER2+), and triple-negative (ER-, PR-, HER2-).

An *in vivo* study found that IQGAP1 expression is significantly elevated in breast cancer cells compared to normal breast cells. Furthermore, endogenous knockdown of IQGAP1 reduces the oncogenicity of MCF-7 cells. The IQGAP1 protein binds to the estrogen receptor (ER), enhancing its transcriptional activity and promoting cancer cell proliferation (38). Overexpression of IQGAP1 increases the binding of GTP to RhoA and RhoC, while IQGAP1 deficiency prevents the growth factor-induced activation of endogenous RhoA and RhoC (18). Another Rho family member, Cdc42, was found to interact with IQGAP1, directly affecting actin cytoskeleton dynamics and filopodia formation (39), thereby promoting breast cancer cell metastasis. As a result, novel small-molecule inhibitors that disrupt their interaction have become a promising area of therapeutic development (40).

IQGAP1 also localizes to the centrosome, where it regulates centrosome size and number by influencing BRCA1 localization, thereby modulating cell proliferation (41). Additionally, a study revealed that Sec1 family domain-containing protein 1 may synergize with IQGAP1, while zinc finger DNA-binding protein (ZN835) was identified as an IQGAP1-binding partner. Together, they promote the metabolism of cancer cells.

Following treatment with the antipsychotic drug pimozide, IQGAP1 forms aggregates with TGF-β1-induced anti-apoptotic factor (TIAF1), thereby promoting apoptosis (42).

#### 3.3.3 Hepatocellular carcinoma

According to 2022 cancer statistics, there were approximately 800,000 newly diagnosed liver cancer cases, accounting for 4.3% of all new cancer cases and ranking sixth globally. Liver cancer-related deaths totaled approximately 750,000, making it the fourth leading cause of cancer-related mortality (32). Liver cancer primarily includes hepatocellular carcinoma (HCC), which accounts for about 75%–85%, and intrahepatic cholangiocarcinoma, which accounts for 10%–15%. The mechanisms underlying HCC development are highly complex, with the expression of IQGAP1 and IQGAP2 believed to play significant roles.

Studies have shown that most human HCC patients exhibit dysregulation in the typical *Wnt/Frizzled signaling* cascade (43), often involving the gene encoding β-catenin (44). IQGAP1 mediates the *Wnt/β-catenin signaling pathway*, promoting cancer metastasis. Furthermore, IQGAP1 is strongly associated with Ras in the signaling network (45). Overexpression of IQGAP1 enhances the growth and metastasis of HCC (27, 46, 47), while IQGAP1-deficient cancer cells are more sensitive to apoptosis. On the other hand, IQGAP2 exerts an inhibitory effect on HCC (48, 49), and the expression levels of IQGAP1 and IQGAP2 influence patient prognosis. High IQGAP1 expression typically indicates poor HCC prognosis (50), whereas the absence of both IQGAP1 and IQGAP2 exhibits a protective mechanism against liver cancer (27).

Jin et al. conducted immunostaining on 33 pairs of HCC tissues and adjacent non-cancerous tissues, revealing a significant correlation between IQGAP1 and β-catenin. IQGAP1 promotes the transcription and nuclear translocation of β-catenin and enhances the expression of β-catenin-mediated Wnt target genes, driving HCC cell migration (51). However, recent studies suggest that co-expression of IQGAP1 and active β-catenin does not show additive or synergistic effects. While IQGAP1 alone or in combination with activated β-catenin cannot promote HCC development *in vivo*, its overexpression significantly accelerates HCC progression (52). Another study found that IQGAP1-shRNA protects normal liver cell function by regulating the expression of IL-8, TGF-β receptors, and apoptosis-related genes (53).

Chronic HBV infection is the most critical risk factor for HCC. It was discovered that the interaction between IQGAP1 and Rho family proteins plays a vital role. In HBV-positive liver cancer cells, IQGAP1 is significantly upregulated compared to HBV-negative cells. IQGAP1 promotes HBV-mediated anti-apoptosis and metastasis through Rac1-dependent ROS accumulation and activation of the *Src/FAK pathway (* 28).

Sorafenib is a drug used to treat advanced HCC, but resistance to it limits its therapeutic success (54). Re-activation of the *PI3K/Akt signaling pathway* contributes to sorafenib resistance. A recent study developed a dual sgRNA plasmid targeting IQGAP1 and FOXM1, integrated into naturally secreted extracellular vesicles for CRISPR delivery. This approach inhibited IQGAP1-mediated *Akt signaling* activation, thereby overcoming sorafenib resistance (55).

Notably, targeted disruption of the mouse Iqgap1 gene does not cause any defects except for late-stage gastric mucosal hyperplasia (56). Therefore, IQGAP1 is considered an essential target for HCC treatment and prognosis prediction.

#### 3.3.4 Pancreatic cancer

Pancreatic cancer is one of the most common gastrointestinal tumors, characterized by its high malignancy and poor overall prognosis, with a 5-year survival rate of only 9%. The current treatment approach for pancreatic cancer remains surgical resection combined with adjuvant chemotherapy. However, due to the insidious onset of pancreatic cancer, most patients are diagnosed at an advanced stage, making treatment outcomes often suboptimal (57). Identifying an effective targeted therapy to improve the prognosis of pancreatic cancer has become a significant challenge.

The mechanism of IQGAP1 in pancreatic cancer has been found to be strongly associated with the *MAPK pathway*. KRAS mutations are considered an initiating event in pancreatic ductal adenocarcinoma (PDAC). KRAS mutations activate downstream pathways, including *MAPK1 and ARL4C signaling*, promoting cell proliferation. KRAS mutations activate ARL4C, which recruits MMP14 via its interaction with IQGAP1, forming invasive pseudopodia that contribute to the uncontrolled invasion and metastasis of pancreatic cancer (58). Additionally, KRAS activation mediates *MAPK signaling* through IQGAP1, facilitating PDAC cell clonal growth and self-renewal (59). Phosphoproteomic analysis of pancreatic cancer tissues revealed significantly elevated phosphorylation levels of IQGAP1, and protein blotting results showed that IQGAP1 phosphorylation is regulated by MAPK1 kinase. *In vitro* experiments using a dual-luciferase reporter assay confirmed that SOX4 binds to MAPK1 and promotes its transcription, establishing the role of the SOX4/MAPK1/IQGAP1 regulatory axis in pancreatic cancer cells (60).

Gemcitabine is the first-line chemotherapeutic drug for pancreatic cancer, but its efficacy is limited by drug resistance. Fructose-1,6-bisphosphatase (FBP1) competes with ERK1/2 for binding to the WW domain of IQGAP1, thereby inhibiting ERK1/2 phosphorylation. A small peptide inhibitor derived from FBP1 can eliminate gemcitabine-induced ERK activation, enhancing its efficacy (61). Similarly, a peptide mimicking the IQGAP1 WW domain can disrupt the interaction between IQGAP1 and ERK, extending the survival time of PDAC mouse models (59).

IQGAP1 promotes pancreatic cancer progression by increasing the levels of DVL2 protein, enhancing the *canonical Wnt/β-catenin pathway*, and driving cell migration, invasion, and epithelial-mesenchymal transition (EMT) (62). The E3 ubiquitin ligase Mindbomb 1 (MIB1), previously found to be highly expressed in pancreatic cancer cells and associated with poor prognosis, has been shown to induce the degradation of the tumor suppressor ST7 through the ubiquitin-proteasome system, thereby increasing IQGAP1 expression and promoting pancreatic cancer migration and invasion (63).

Pro-inflammatory cytokines are also drivers of tumor metastasis and invasion. *IL-6 signaling* via the *STAT3 pathway* is integrated by IQGAP1 into CDC42, promoting actin remodeling and the formation of pre-migratory filopodia, thereby driving metastasis and invasion. IQGAP1 knockout blocks *IL-6-dependent CDC42 signaling*, further validating IQGAP1 as an information integration platform for STAT3 and CDC42 (64).

#### 3.3.5 Gastric cancer

In 2022, there were over 968,000 new cases of gastric cancer and nearly 660,000 deaths, ranking fifth globally in both incidence and mortality (32). Recent studies have shown that the onset of gastric cancer is trending toward younger age groups (65). The mechanisms by which IQGAP1 participates in the progression of gastric cancer are complex and not fully understood. Research indicates that various biomolecules interact with IQGAP1, enhancing the metastasis and invasion of gastric cancer cells. During gastric cancer progression, IQGAP1 directly interacts with RhoC, enhancing the expression of cyclins E and D1, indirectly affecting the G1/S transition and promoting cell proliferation (66).

IQGAP1 also acts as a scaffold protein regulating alternative splicing (AS) of different gene subgroups in gastric cancer cells. It participates in AS regulation that significantly impacts mitochondrial respiration, altering the activity of mitochondrial respiratory chain complex I (CI), thereby conferring proliferative advantages, drug resistance, and other malignant characteristics to gastric cancer cells (67).

Recent studies have discovered other molecular partners involved in IQGAP1-mediated migration and invasion of gastric cancer cells. One study found an interaction between pepsinogen (PGC) and IQGAP1, with a significant negative correlation between their expressions in gastric cancer cells. Overexpression of PGC markedly reduces the half-life of IQGAP1 and inhibits the Rho-GTPase regulatory network, thereby suppressing cell migration and proliferation (68). Research has demonstrated the potential of PGC in gastric cancer screening and prevention (69), making the PGC-IQGAP1 interaction a potential research direction for improving gastric cancer prognosis.

ASAP1 (Arf GTPase-activating protein 1), a marker of malignant tumors, regulates the cytoskeleton to promote tumor cell metastasis and invasion (70). *In vivo* experiments revealed a strong correlation between IQGAP1 and ASAP1. When IQGAP1 was knocked down, ASAP1 lost its ability to promote cell migration and invasion. Conversely, ASAP1 overexpression significantly upregulated IQGAP1 protein levels by inhibiting ubiquitin-mediated degradation of IQGAP1 (71), enhancing CDC42 activity and promoting cell migration. The study also found that ASAP1, through IQGAP1-activated CDC42, may upregulate *EGFR-MAPK signaling*, contributing to resistance to common gastric cancer chemotherapy drugs such as 5-fluorouracil and oxaliplatin (72).

Menin has been found to interact with IQGAP1, enhancing cell adhesion while reducing cell metastasis and invasion (73). Recent research indicates that menin inhibits gastric cancer cell proliferation by suppressing IQGAP1 and downregulating *PI3K* and *NF-κB* expression (74).

In recent years, molecules such as Rho family proteins, PGC, ASAP1, and menin have been shown to interact with IQGAP1, either enhancing or inhibiting its activity. Further elucidating the mechanisms of IQGAP1 in gastric cancer progression and incorporating these findings into targeted therapies to enable early detection and improved prognosis for gastric cancer patients will remain a critical area of research.

#### 3.3.6 Colorectal cancer

With healthier lifestyles and the gradual lowering of the age for colorectal cancer screening via colonoscopy, the incidence of colorectal cancer has begun to decline in many high-incidence countries. However, colorectal cancer remains the third most common cancer worldwide, with its mortality rate ranking second (32, 75). IQGAP1 is implicated in the development and progression of colorectal cancer and is highly associated with cellular invasiveness, though the precise mechanisms are not yet fully understood. IQGAP1’s involvement in colorectal cancer is closely related to the *ERK pathway*. Ding et al. found that sustained activation of the *MAPK/ERK signaling pathway* is commonly observed in CRC patients with poor prognosis (76), and IQGAP1 activates ERK phosphorylation, further demonstrating that CRC cell growth, migration, and tumorigenesis are influenced by IQGAP1 (77). Another *in vivo* study revealed that PLS1 (actin bundling protein 1) promotes CRC cell metastasis by regulating the *IQGAP1/Rac1/ERK signaling pathway (* 78).

The tumor microenvironment (TME) plays a crucial role in cancer development and progression, participating in immune cell activation and recruitment, angiogenesis, and extracellular matrix remodeling. It is closely associated with patient prognosis and serves as an important target for therapeutic research (79). IQGAP1 interacts with MYL9 and acts on the *ERK1/2 pathway* to regulate cancer-associated fibroblasts (CAFs), which secrete CCL2 and TGF-β1, thereby influencing the tumor microenvironment and patient outcomes (80). Familial genetic mutations such as CGN c.3560C>T have been identified as susceptibility factors for colorectal cancer. One study found that CGN c.3560C>T triggers IQGAP1 overexpression and activates Rac1-dependent EMT, promoting the initiation and metastasis of colorectal cancer (81).

In terms of treatment, the primary approach for colorectal cancer remains surgery combined with chemotherapy. However, the effectiveness of chemotherapy is limited by drug resistance. For instance, CRC resistant to the chemotherapy drug Vemurafenib exhibits increased invasiveness, likely due to the reactivation of the *MAPK/ERK pathway (* 82). A recent study confirmed this, showing that ARF1-IQGAP1 interactions promote *ERK pathway signaling*, playing a critical role in Vemurafenib resistance and cancer metastasis. A small-molecule inhibitor (LY2839219) significantly suppressed CRC metastasis by disrupting this interaction (29). Furthermore, stable transfection of IQGAP1-shRNA reduced IQGAP1 expression, leading to decreased migration ability in CRC cells (83).

The development, metastasis, and clinical drug resistance of colorectal cancer are all closely related to the *IQGAP1/ERK pathway*. IQGAP1 interacts with various molecules to regulate the *ERK pathway*, making it a promising target for therapeutic strategies. Targeted therapies focusing on the *ERK signaling pathway* and IQGAP1 provide new directions for the treatment and prognosis of colorectal cancer.

#### 3.3.7 Glioma

Gliomas are the most common primary malignant intracranial tumors, and patients generally have a poor prognosis. Gliomas are classified into pathological grades (WHO CNS5): Grade 1, typically low-grade, includes pilocytic astrocytomas; Grade 2 is low-grade but diffusely infiltrative; Grade 3 includes anaplastic astrocytomas and anaplastic ependymomas; and Grade 4 includes glioblastoma. Grades 1–2 are considered low-grade gliomas, while Grades 3–4 are high-grade gliomas. Molecular pathological classification based on tumor genetics also plays a critical role in determining clinical prognosis (84).

The molecular targets and pathways involved in glioblastoma include the epidermal growth factor receptor (EGFR)-mediated *PI3K/Akt* and *MAPK pathways*, the PTEN-regulated *mTOR/PI3K/Akt pathway*, *NF-Kb* and *the JAK/STAT pathway (* 85, 86). Among these known molecular targets and pathways, IQGAP1 serves as an upstream molecule or molecular partner, playing an indispensable role in regulating multiple pathways. *In vivo* experiments have validated the role of IQGAP1, demonstrating that knocking down IQGAP1 expression arrests the proliferation of low-grade glioma cells, with most cells halting in the G0/G1 phase. These cells also exhibited greater sensitivity to the chemotherapeutic agent cisplatin (87).

The expression of IQGAP1 is positively correlated with CDC42, Ih promotes tumor malignancy (88). New molecules have been identified in relation to this mechanism. For instance, TRIM56, a ubiquitin ligase, interacts with IQGAP1 to increase its K63-linked ubiquitination, preventing its degradation and enhancing CDC42 expression (89). TRIM56 and activated CDC42 co-localize in focal adhesions, promoting actin cytoskeleton reorganization and regulating cell invasion and migration (90).

The primary treatment for gliomas Is surgical resection, combined with radiotherapy and chemotherapy. However, chemoresistance limits treatment efficacy, and the blood-brain barrier restricts the use of small-molecule drugs. Molecular markers have significant clinical value for personalized treatment and prognosis evaluation. To overcome the limitations of molecular therapies, studies have found that certain peptides (such as insulin receptor, transferrin, and EGFR peptides) can cross the blood-brain barrier through receptor-mediated endocytosis and bind to IQGAP1 (91), making IQGAP1 a promising target for small-molecule drug therapies.

miR-124, which is abundantly expressed in normal brain tissue but significantly downregulated in glioma cells, has been shown to target IQGAP1 and regulate downstream signaling to inhibit glioma cell migration and invasion (92).Additionally, the traditional Chinese medicine compound shikonin has been found to reduce IQGAP1 expression and mTOR phosphorylation, potentially modulating *the mTOR/Akt pathway*. This regulation arrests the cell cycle in the G1 phase, thereby inhibiting glioma cell proliferation (93).

The above biomolecules Interact with IQGAP1 to regulate glioma cell proliferation. However, challenges such as the blood-brain barrier and drug resistance remain significant hurdles. Developing novel targeted therapies through IQGAP1 continues to be a formidable but promising research endeavor (**Table 1**).

**Table 1:** The specific functions of IQGAP1.

Disease	Related pathways/molecules	Effect on disease	Potential therapeutic directions	Ref
Head and Neck Cancer	PI3K/AKT/mTOR pathway; HPV/Rac1/CDC42	Drives aberrant splicing events; Enhances HPV oncoprotein-mediated invasion	Targeting IQGAP1/PI3K signaling axis	(33–36)
Breast Cancer	RhoA/RhoC/CDC42; ER transcriptional regulation; BRCA1 centrosome localization	Activates actin reorganization; Enhances estrogen receptor activity; Regulates cell cycle	Small-molecule inhibitors (blocking IQGAP1-CDC42 interaction)	(18, 38–41)
Hepatocellular Carcinoma	Wnt/β-catenin pathway; Rac1/ROS/Src/FAK pathway; PI3K/Akt pathway	Promotes β-catenin transcription and nuclear translocation; Mediates anti-apoptosis and metastasis; Induces sorafenib resistance	CRISPR-based IQGAP1/FOXM1 delivery system ; IQGAP1-shRNA to suppress metastasis	(28, 51–53)
Pancreatic Cancer	KRAS/MAPK pathway; SOX4/MAPK1 axis; IL-6/STAT3/CDC42	Promoves invasive pseudopodia formation ;Enhances EMT ; Mediates gemcitabine resistance	FBP1-derived peptide blocking ERK activation; Targeting MIB1-IQGAP1 axis	(58–61, 63, 64)
Gastric Cancer	RhoC/cell cycle proteins; ASAP1/CDC42; PGC negative regulation	Accelerates G1/S phase transition; Inhibits ubiquitination degradation; Modulates mitochondrial respiratory chain	PGC overexpression; Targeting ASAP1-IQGAP1 axis; Menin inhibition of PI3K/NF-κB	(66–68, 71, 72, 74)
Colorectal Cancer	MAPK/ERK pathway; PLS1/Rac1/ERK axis; CCL2/TGF-β1 (tumor microenvironment)	Activates ERK phosphorylation; Promotes CAFs to secrete pro-tumor factors	Targeting ARF1-IQGAP1 (LY2839219); IQGAP1 knockdown to inhibit invasion	(77–80)
Glioma	CDC42/TRIM56 ubiquitination; miR-124 targeting inhibition; mTOR/Akt pathway	Drives malignant progression; Reduces chemotherapy sensitivity	miR-124 mimics ; Shikonin inhibits mTOR ; Blood-brain barrier-penetrating drug delivery	(89–93)

## 4 Role of IQGAP1 in immune regulation

### 4.1 Interaction of IQGAP1 with immune receptors

#### 4.1.1 cGAS-STING pathway and NLRP3 activation

IQGAP1 plays a role in immune regulation by promoting the release of mitochondrial DNA (mtDNA) into the cytoplasm, which activates the *cGAS-STING pathway*. This activation leads to the subsequent NLRP3 inflammasome-mediated pyroptosis in endothelial cells. Specifically, in atherosclerotic mice, silencing IQGAP1 reduced mtDNA release and suppressed the expression of DNA receptors, thus demonstrating IQGAP1’s role in modulating inflammation through mitochondrial stress (94).

#### 4.1.2 CXCR4 and chemotaxis

IQGAP1 is involved in immune cell migration through its interaction with CXCR4, a chemokine receptor. It is essential for the surface expression and signaling of CXCR4 in neutrophils, influencing cell migration and immune responses. IQGAP1’s scaffold function regulates CXCR4 transport and recycling, playing a key role in immune cell trafficking during inflammation (21).

#### 4.1.3 CD11c and neutrophil function

IQGAP1 interacts directly with CD11c, an adhesion molecule critical for neutrophil function. In neutrophils, IQGAP1 modulates ROS generation, phagocytosis, and extracellular trap formation, highlighting its involvement in neutrophil activation and immune defense (95).

#### 4.1.4 Rap1 and immune signaling

IQGAP1 binds to Rap1, a small GTPase involved in cell adhesion and migration. This interaction is crucial for regulating immune cell migration and function. By influencing Rap1 activation, IQGAP1 modulates processes such as cytokine secretion and immune cell adhesion, which are vital for proper immune responses (96).

### 4.2 IQGAP1 in immune diseases

#### 4.2.1 IQGAP1 and inflammatory diseases

**Figure 3:**
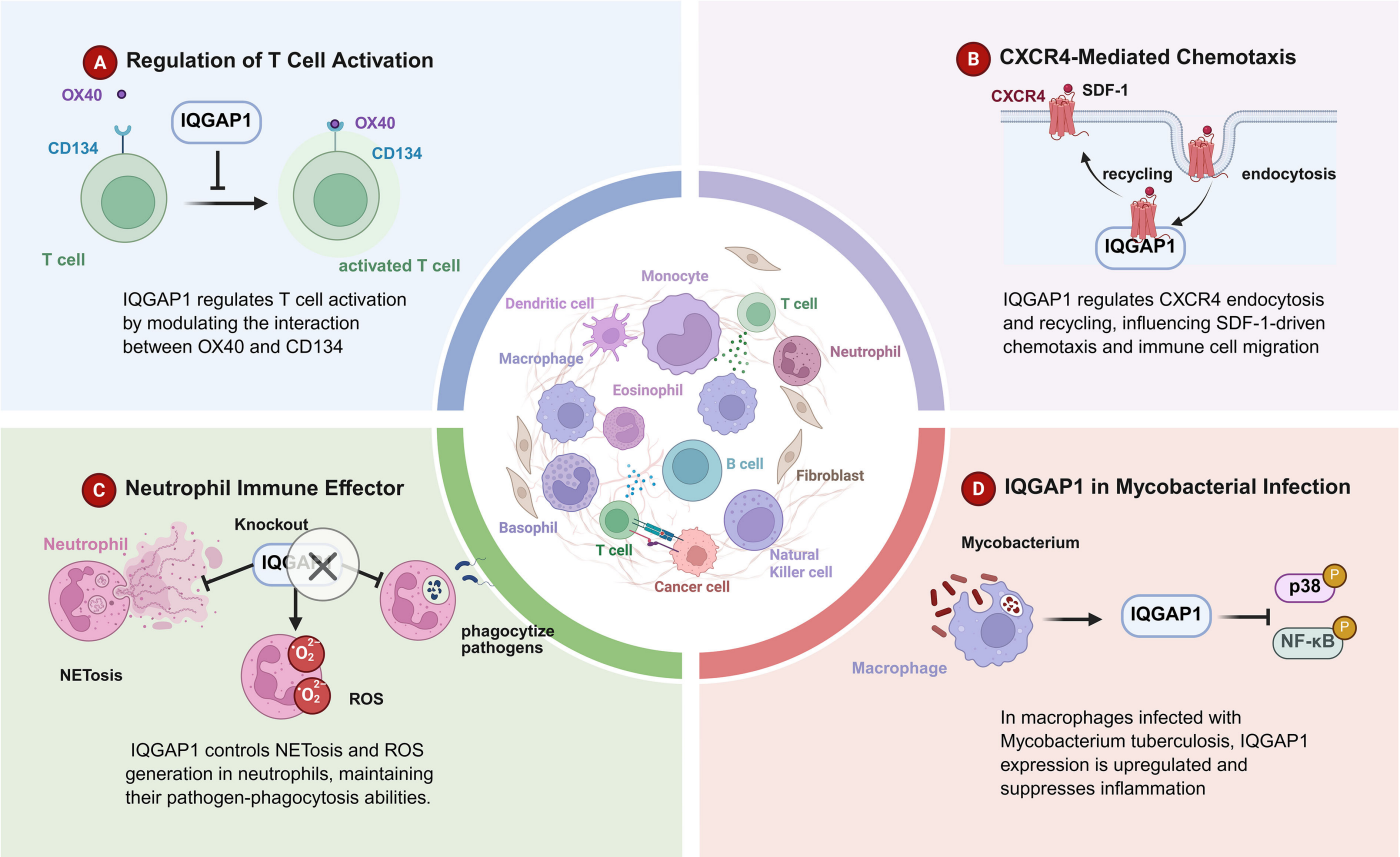
IQGAP1 in immune regulation. This figure outlines the diverse roles of IQGAP1 in regulating immune cell function across different contexts. **(A)** Regulation of T Cell Activation: IQGAP1 modulates T cell activation by influencing the interaction between the costimulatory receptors OX40 and CD134, promoting T cell activation. **(B)** CXCR4-Mediated Chemotaxis: IQGAP1 regulates the endocytosis and recycling of the CXCR4 receptor, which is critical for SDF-1-driven chemotaxis and immune cell migration, contributing to effective immune responses. **(C)** Neutrophil Immune Effector Function: IQGAP1 controls the processes of NETosis and ROS generation in neutrophils, which are essential for maintaining their pathogen-phagocytosis abilities. Neutrophil dysfunction following IQGAP1 knockout impairs immune responses. **(D)** IQGAP1 in Mycobacterial Infection: In macrophages infected with Mycobacterium tuberculosis, IQGAP1 expression is upregulated and plays a role in suppressing inflammation through the p38 and NF-κB signaling pathways.

IQGAP1 interacts with the OX40 receptor (CD134), a co-stimulatory molecule essential for T cell immune responses, and regulates T cell proliferation and cytokine secretion. In the absence of IQGAP1, OX40-induced T cell proliferation and cytokine secretion are enhanced, indicating its role as a negative regulator of T cell activation. Moreover, IQGAP1-deficient mice exhibit exacerbated experimental autoimmune encephalomyelitis (EAE), further emphasizing its critical role in limiting T cell-mediated inflammation (9) (**Figure 3**).

#### 4.2.2 IQGAP1 in neutrophil function

IQGAP1 is crucial for neutrophil function, especially in processes like ROS generation, phagocytosis, and the formation of extracellular traps. IQGAP1 deficiency leads to impaired neutrophil maturation and functionality, underscoring its importance in neutrophil-mediated immune responses (95).

#### 4.2.3 IQGAP1 in leukocyte transmigration

IQGAP1 plays an essential role in the transendothelial migration (TEM) of leukocytes, a critical step in the immune response to inflammation. Its absence disrupts the recruitment of leukocytes to sites of inflammation by affecting the localization and movement of the lateral border recycling compartment (LBRC) in endothelial cells (97).

#### 4.2.4 IQGAP1 in tuberculosis

During mycobacterial infections like TB, IQGAP1 modulates the inflammatory response. It helps control *MKK3 signaling*, limiting NF-κBp65 nuclear translocation and the activation of pro-inflammatory pathways. IQGAP1’s regulation of the immune response is critical for managing granuloma formation and reducing the tissue damage associated with TB (98).

## 5 Role of IQGAP1 in metabolic diseases

Recent research has shed light on the complex involvement of IQGAP1 in various metabolic diseases. Here, we explore the potential mechanisms through which IQGAP1 influences metabolic disorders, with a focus on its regulation of key metabolic pathways and its implications in diseases such as diabetes, obesity, cardiovascular disease, non-alcoholic fatty liver disease (NAFLD), and osteoporosis.

### 5.1 IQGAP1 and the AMPK signaling pathway

One of the most well-characterized functions of IQGAP1 is its involvement in regulating the *AMP-activated protein kinase (AMPK) signaling pathway*. *AMPK* acts as a cellular energy sensor, maintaining energy homeostasis by activating catabolic pathways and inhibiting anabolic pathways when cellular energy levels are low. Recent studies have shown that IQGAP1 directly interacts with the α1 subunit of *AMPK*, promoting its activity. In the absence of IQGAP1, *AMPK* activation is impaired, leading to alterations in glucose homeostasis and lipid metabolism, key features of metabolic diseases like type 2 diabetes. Therefore, the IQGAP1-AMPK axis presents a potential target for therapeutic interventions in metabolic disorders (99).

### 5.2 IQGAP1 in diabetic kidney disease

In diabetic kidney disease (DKD), a complication of long-term diabetes, IQGAP1 has been implicated in the progression of renal injury. High glucose conditions induce the overexpression of IQGAP1, which interacts with the renin-angiotensin system (RAS) to modulate renal function. Specifically, IQGAP1 regulates the internalization of renin in podocytes, contributing to the pathogenesis of kidney fibrosis and glomerulosclerosis. These findings suggest that IQGAP1 may exacerbate renal damage in diabetes and could be a potential therapeutic target for preventing or slowing DKD progression (4).

### 5.3 IQGAP1 and osteoporosis

IQGAP1 has been implicated in the regulation of bone metabolism, particularly in bone formation. Osteoporosis, characterized by reduced bone mass and increased fracture risk, is a metabolic bone disease influenced by both genetic and environmental factors. IQGAP1 regulates osteoblast differentiation and bone mineralization through its interaction with the *SMAD2/3 signaling pathway*, which is central to *transforming growth factor-beta (TGF-β) signaling* involved in bone formation. Disruptions in IQGAP1 function result in abnormal bone turnover, providing a novel perspective on how metabolic dysregulation can contribute to bone fragility and osteoporosis. Thus, IQGAP1 may serve as a promising target for developing therapeutic strategies aimed at improving bone health in osteoporotic patients (100).

### 5.4 Chronic pain

In addition to playing a crucial role in tumor progression, IQGAP1 is involved in other biological processes. Recent studies suggest that IQGAP1 may have a role in the development of pain. A genome-wide association study reported that two loci, IQGAP1 and CRTC3, are significantly associated with chronic postoperative pain three months after surgery (101). This suggests that IQGAP1 could be a key genetic factor in pain susceptibility. Another study revealed that IQGAP1 participates in the process of chronic pain. Inflammatory signals, such as PKA and Ca²^+^, increase the binding of TRPA1 (a nociceptor) to IQGAP1. IQGAP1 anchors Cdc42 and carries it near TRPA1, acting as a molecular switch to facilitate the transport of Ca²^+^ and PKA channels, thereby sensitizing TRPA1. This molecular mechanism may explain how IQGAP1 amplifies nociceptive signals during chronic pain conditions. Moreover, evidence suggests that IQGAP1’s role in pain extends beyond nociception, potentially influencing the persistence of pain by regulating neural plasticity and synaptic changes in pain processing areas of the brain (102).

Additionally, a new study reported that IQGAP1 is involved in the addiction and cognitive side effects of opioid drugs such as morphine. In comparative experiments and luciferase assays involving morphine-dependent and non-dependent patients, researchers found that morphine-dependent patients exhibited high levels of miR-124 and low levels of IQGAP1. This suggests that miR-124-mediated downregulation of IQGAP1 could be a key factor in the cognitive deficits associated with opioid addiction. It is likely that IQGAP1 may influence synaptic plasticity and neuroinflammation, both of which play roles in addiction and cognitive function (103).

## 6 Conclusion

IQGAP1 is a versatile scaffold protein that plays a critical role in regulating cellular processes including cytoskeletal dynamics, signal transduction, and cellular interactions, thereby influencing fundamental cellular behaviors such as migration, proliferation, and differentiation. It serves as a key intermediary in a variety of signaling networks, interacting with small GTPases, receptor tyrosine kinases, and other regulatory proteins to control essential cellular functions. Beyond its roles in normal cellular physiology, IQGAP1 has been implicated in a range of pathological conditions, including various cancers, immune diseases, metabolic disorders, and cardiovascular conditions. Its multifaceted nature makes it an attractive target for therapeutic intervention.

In cancer, IQGAP1 is involved in promoting tumor growth, metastasis, and resistance to chemotherapy and targeted therapies. As cancer therapies face the growing challenge of drug resistance, targeting IQGAP1 could offer a novel strategy for overcoming this hurdle. In addition to its role in cancer, IQGAP1 is implicated in immune system modulation, particularly in autoimmune diseases where its regulatory functions may affect the development and progression of inflammation and immune responses. Furthermore, IQGAP1’s role in cellular metabolism and energy balance suggests its potential as a target for metabolic disorders, such as obesity and diabetes, where its involvement in signaling pathways related to insulin resistance and cell survival is critical.

While our understanding of IQGAP1’s biology has advanced, important questions remain. These include how it achieves signaling specificity in diverse tissues, how regulatory mechanisms such as post-translational modifications shape its context-dependent functions, and whether its pathological roles can be selectively targeted without compromising physiological ones.

Recent interest in modulating scaffold proteins has opened avenues for targeting IQGAP1’s disease-specific interactions. Although it lacks intrinsic enzymatic activity, emerging tools such as interface inhibitors, molecular degraders, and peptidomimetics may offer routes to selectively disrupt pathogenic complexes. Therapeutic development in this space remains early-stage, but shows growing potential—especially if coupled with biomarker-guided patient selection and insights into disease context.

Translating these findings into clinical applications will require bridging several methodological gaps. These include the generation of tissue-specific and inducible IQGAP1 knockout models, high-resolution structural analyses of its functional domains in disease states, and systematic mapping of its interactome using advanced spatial proteomics. Moreover, identifying robust biomarkers of IQGAP1 dependency will be essential for stratifying patients and optimizing therapeutic efficacy.

In conclusion, IQGAP1 represents a versatile regulatory hub with broad translational potential. As mechanistic insights deepen and novel targeting strategies mature, IQGAP1-based interventions may emerge as powerful tools in addressing unmet clinical needs across cancer, metabolic disorders, immune dysregulation, and beyond.

## References

[1] Brown MD Sacks DB. IQGAP1 in cellular signaling: bridging the GAP. Trends Cell Biol. (2006) 16:242–9. doi: 10.1016/j.tcb.2006.03.002, PMID: 16595175

[2] McNulty DE Li Z White CD Sacks DB Annan RS. MAPK scaffold IQGAP1 binds the EGF receptor and modulates its activation. J Biol Chem. (2011) 286:15010–21. doi: 10.1074/jbc.M111.227694, PMID: 21349850 PMC3083173

[3] Lehtonen S Ryan JJ Kudlicka K Iino N Zhou H Farquhar MG. Cell junction-associated proteins IQGAP1, MAGI-2, CASK, spectrins, and alpha-actinin are components of the nephrin multiprotein complex. Proc Natl Acad Sci U S A. (2005) 102:9814–9. doi: 10.1073/pnas.0504166102, PMID: 15994232 PMC1175008

[4] Liu Y Su H Ma C Ji D Zheng X Wang P. IQGAP1 mediates podocyte injury in diabetic kidney disease by regulating nephrin endocytosis. Cell Signal. (2019) 59:13–23. doi: 10.1016/j.cellsig.2019.03.009, PMID: 30857827

[5] Balenci L Saoudi Y Grunwald D Deloulme JC Bouron A Bernards A. IQGAP1 regulates adult neural progenitors *in vivo* and vascular endothelial growth factor-triggered neural progenitor migration *in vitro* . J Neurosci. (2007) 27:4716–24. doi: 10.1523/JNEUROSCI.0830-07.2007, PMID: 17460084 PMC6672986

[6] Yamaoka-Tojo M Ushio-Fukai M Hilenski L Dikalov SI Chen YE Tojo T. IQGAP1, a novel vascular endothelial growth factor receptor binding protein, is involved in reactive oxygen species–dependent endothelial migration and proliferation. Circ Res. (2004) 95:276–83. doi: 10.1161/01.RES.0000136522.58649.60, PMID: 15217908

[7] Sbroggiò M Carnevale D Bertero A Cifelli G De Blasio E Mascio G. IQGAP1 regulates ERK1/2 and AKT signalling in the heart and sustains functional remodelling upon pressure overload. Cardiovasc Res. (2011) 91:456–64. doi: 10.1093/cvr/cvr103, PMID: 21493702 PMC3294280

[8] Hedman AC Smith JM Sacks DB. The biology of IQGAP proteins: beyond the cytoskeleton. EMBO Rep. (2015) 16:427–46. doi: 10.15252/embr.201439834, PMID: 25722290 PMC4388610

[9] Okuyama Y Nagashima H Ushio-Fukai M Croft M Ishii N So T. IQGAP1 restrains T-cell cosignaling mediated by OX40. FASEB J. (2020) 34:540–54. doi: 10.1096/fj.201900879RR, PMID: 31914585

[10] Jufvas Å Rajan MR Jönsson C Strålfors P Turkina MV. Scaffolding protein IQGAP1: an insulin-dependent link between caveolae and the cytoskeleton in primary human adipocytes? Biochem J. (2016) 473:3177–88. doi: 10.1042/BCJ20160581, PMID: 27458251

[11] Chawla B Hedman AC Sayedyahossein S Erdemir HH Li Z Sacks DB. Absence of IQGAP1 protein leads to insulin resistance. J Biol Chem. (2017) 292:3273–89. doi: 10.1074/jbc.M116.752642, PMID: 28082684 PMC5336162

[12] Zhou H Yao C Bian A Qian J Zhao X Zhao Y. The Ras GTPase-activating-like protein IQGAP1 is downregulated in human diabetic nephropathy and associated with ERK1/2 pathway activation. Mol Cell Biochem. (2014) 391:21–5. doi: 10.1007/s11010-014-1982-x, PMID: 24488174

[13] Smith JM Hedman AC Sacks DB. IQGAPs choreograph cellular signaling from the membrane to the nucleus. Trends Cell Biol. (2015) 25:171–84. doi: 10.1016/j.tcb.2014.12.005, PMID: 25618329 PMC4344846

[14] Roy M Li Z Sacks DB. IQGAP1 is a scaffold for mitogen-activated protein kinase signaling. Mol Cell Biol. (2005) 25:7940–52. doi: 10.1128/MCB.25.18.7940-7952.2005, PMID: 16135787 PMC1234344

[15] Choi S Hedman AC Sayedyahossein S Thapa N Sacks DB Anderson RA. Agonist-stimulated phosphatidylinositol-3,4,5-trisphosphate generation by scaffolded phosphoinositide kinases. Nat Cell Biol. (2016) 18:1324–35. doi: 10.1038/ncb3441, PMID: 27870828 PMC5679705

[16] Liang Z Yang Y He Y Yang P Wang X He G. SUMOylation of IQGAP1 promotes the development of colorectal cancer. Cancer Lett. (2017) 411:90–9. doi: 10.1016/j.canlet.2017.09.046, PMID: 28987385

[17] Chen Y Mei J Zhang P Liu J Chen L Wu L. IQGAP1 is positively correlated with PD-L1 and regulates its expression via mediating STAT proteins phosphorylation. Int Immunopharmacol. (2022) 108:108897. doi: 10.1016/j.intimp.2022.108897, PMID: 35729832

[18] Erdemir HH Li Z Sacks DB. IQGAP1 binds to estrogen receptor-α and modulates its function. J Biol Chem. (2014) 289:9100–12. doi: 10.1074/jbc.M114.553511, PMID: 24550401 PMC3979404

[19] Thines L Li Z Sacks DB. IQGAP1 is a phosphotyrosine-regulated scaffold for SH2-containing proteins. Cells. (2023) 12:14–15. doi: 10.3390/cells12030483, PMID: 36766826 PMC9913818

[20] Malarkannan S Awasthi A Rajasekaran K Kumar P Schuldt KM Bartoszek A. IQGAP1: a regulator of intracellular spacetime relativity. J Immunol. (2012) 188:2057–63. doi: 10.4049/jimmunol.1102439, PMID: 22345702 PMC3286039

[21] Bamidele AO Kremer KN Hirsova P Clift IC Gores GJ Billadeau DD. IQGAP1 promotes CXCR4 chemokine receptor function and trafficking via EEA-1+ endosomes. J Cell Biol. (2015) 210:257–72. doi: 10.1083/jcb.201411045, PMID: 26195666 PMC4508899

[22] White CD Erdemir HH Sacks DB. IQGAP1 and its binding proteins control diverse biological functions. Cell Signal. (2012) 24:826–34. doi: 10.1016/j.cellsig.2011.12.005, PMID: 22182509 PMC3268868

[23] Cerutti C Lucotti S Menendez ST Reymond N Garg R Romero IA. IQGAP1 and NWASP promote human cancer cell dissemination and metastasis by regulating β1-integrin via FAK and MRTF/SRF. Cell Rep. (2024) 43:113989. doi: 10.1016/j.celrep.2024.113989, PMID: 38536816

[24] Kozlova I Ruusala A Voytyuk O Skandalis SS Heldin P. IQGAP1 regulates hyaluronan-mediated fibroblast motility and proliferation. Cell Signal. (2012) 24:1856–62. doi: 10.1016/j.cellsig.2012.05.013, PMID: 22634185

[25] Roy M Li Z Sacks DB. IQGAP1 binds ERK2 and modulates its activity. J Biol Chem. (2004) 279:17329–37. doi: 10.1074/jbc.M308405200, PMID: 14970219

[26] Quinn NP García-Gutiérrez L Doherty C von Kriegsheim A Fallahi E Sacks DB. IQGAP1 is a scaffold of the core proteins of the hippo pathway and negatively regulates the pro-apoptotic signal mediated by this pathway. Cells. (2021) 10:4–13. doi: 10.3390/cells10020478, PMID: 33672268 PMC7926663

[27] Chen F Zhu HH Zhou LF Wu SS Wang J Chen Z. IQGAP1 is overexpressed in hepatocellular carcinoma and promotes cell proliferation by Akt activation. Exp Mol Med. (2010) 42:477–83. doi: 10.3858/emm.2010.42.7.049, PMID: 20530982 PMC2912475

[28] Mo CF Li J Yang SX Guo HJ Liu Y Luo XY. IQGAP1 promotes anoikis resistance and metastasis through Rac1-dependent ROS accumulation and activation of Src/FAK signalling in hepatocellular carcinoma. Br J Cancer. (2020) 123:1154–63. doi: 10.1038/s41416-020-0970-z, PMID: 32632148 PMC7525663

[29] Hu HF Gao GB He X Li YY Li YJ Li B. Targeting ARF1-IQGAP1 interaction to suppress colorectal cancer metastasis and vemurafenib resistance. J Adv Res. (2023) 51:135–47. doi: 10.1016/j.jare.2022.11.006, PMID: 36396045 PMC10491971

[30] Li X Zhao X Su X Wen J Yang S Qin Y. IQGAP1 overexpression attenuates chemosensitivity through YAP-mediated ferroptosis inhibition in esophageal squamous cell cancer cells. Arch Biochem Biophys. (2024) 758:110064. doi: 10.1016/j.abb.2024.110064, PMID: 38897534

[31] Holck S Nielsen HJ Hammer E Christensen IJ Larsson LI. IQGAP1 in rectal adenocarcinomas: localization and protein expression before and after radiochemotherapy. Cancer Lett. (2015) 356:556–60. doi: 10.1016/j.canlet.2014.10.005, PMID: 25305455

[32] Bray F Laversanne M Sung H Ferlay J Siegel RL Soerjomataram I. Global cancer statistics 2022: GLOBOCAN estimates of incidence and mortality worldwide for 36 cancers in 185 countries. CA Cancer J Clin. (2024) 74:229–63. doi: 10.3322/caac.21834, PMID: 38572751

[33] Wei T Choi S Buehler D Lee D Ward-Shaw E Anderson RA. Role of IQGAP1 in papillomavirus-associated head and neck tumorigenesis. Cancers (Basel). (2021) 13:9–12. doi: 10.3390/cancers13092276, PMID: 34068608 PMC8126105

[34] Muehlbauer LK Wei T Shishkova E Coon JJ Lambert PF. IQGAP1 and RNA splicing in the context of head and neck via phosphoproteomics. J Proteome Res. (2022) 21:2211–23. doi: 10.1021/acs.jproteome.2c00309, PMID: 35980772 PMC9833422

[35] Wei T Choi S Buehler D Anderson RA Lambert PF. A PI3K/AKT scaffolding protein, IQ motif-containing GTPase associating protein 1 (IQGAP1), promotes head and neck carcinogenesis. Clin Cancer Res. (2020) 26:301–11. doi: 10.1158/1078-0432.CCR-19-1063, PMID: 31597661 PMC6942630

[36] Morgan EL Macdonald A. Autocrine STAT3 activation in HPV positive cervical cancer through a virus-driven Rac1-NFκB-IL-6 signalling axis. PloS Pathog. (2019) 15:e1007835. doi: 10.1371/journal.ppat.1007835, PMID: 31226168 PMC6608985

[37] Deshmukh J Pofahl R Pfister H Haase I. Deletion of epidermal Rac1 inhibits HPV-8 induced skin papilloma formation and facilitates HPV-8- and UV-light induced skin carcinogenesis. Oncotarget. (2016) 7:57841–50. doi: 10.18632/oncotarget.11069, PMID: 27506937 PMC5295394

[38] Casteel DE Turner S Schwappacher R Rangaswami H Su-Yuo J Zhuang S. Rho isoform-specific interaction with IQGAP1 promotes breast cancer cell proliferation and migration. J Biol Chem. (2012) 287:38367–78. doi: 10.1074/jbc.M112.377499, PMID: 22992742 PMC3488105

[39] Nobes CD Hall A. Rho, rac, and cdc42 GTPases regulate the assembly of multimolecular focal complexes associated with actin stress fibers, lamellipodia, and filopodia. Cell. (1995) 81:53–62. doi: 10.1016/0092-8674(95)90370-4, PMID: 7536630

[40] Sayedyahossein S Smith J Barnaeva E Li Z Choe J Ronzetti M. Discovery of small molecule inhibitors that effectively disrupt IQGAP1-Cdc42 interaction in breast cancer cells. Sci Rep. (2022) 12:17372. doi: 10.1038/s41598-022-21342-w, PMID: 36253497 PMC9576799

[41] Osman MA Antonisamy WJ Yakirevich E. IQGAP1 control of centrosome function defines distinct variants of triple negative breast cancer. Oncotarget. (2020) 11:2493–511. doi: 10.18632/oncotarget.v11i26, PMID: 32655836 PMC7335670

[42] Iyer VJ Osman MA. The antipsychotic drug haldol modulates IQGAP1-signaling and inhibits cell proliferation in triple negative breast cancer cell lines. MicroPubl Biol. (2023) 2023:1–2. doi: 10.17912/micropub.biology.000823, PMID: 37215640 PMC10199339

[43] Lee HC Kim M Wands JR. Wnt/Frizzled signaling in hepatocellular carcinoma. Front Biosci. (2006) 11:1901–15. doi: 10.2741/1933, PMID: 16368566

[44] de La Coste A Romagnolo B Billuart P Renard CA Buendia MA Soubrane O. Somatic mutations of the beta-catenin gene are frequent in mouse and human hepatocellular carcinomas. Proc Natl Acad Sci U S A. (1998) 95:8847–51. doi: 10.1073/pnas.95.15.8847, PMID: 9671767 PMC21165

[45] Zoheir KM Abd-Rabou AA Harisa GI Ashour AE Ahmad SF Attia SM. Gene expression of IQGAPs and Ras families in an experimental mouse model for hepatocellular carcinoma: a mechanistic study of cancer progression. Int J Clin Exp Pathol. (2015) 8:8821–31., PMID: 26464624 PMC4583856

[46] White CD Khurana H Gnatenko DV Li Z Odze RD Sacks DB. IQGAP1 and IQGAP2 are reciprocally altered in hepatocellular carcinoma. BMC Gastroenterol. (2010) 10:125. doi: 10.1186/1471-230X-10-125, PMID: 20977743 PMC2988069

[47] Schmidt VA. Watch the GAP: emerging roles for IQ motif-containing GTPase-activating proteins IQGAPs in hepatocellular carcinoma. Int J Hepatol. (2012) 2012:958673. doi: 10.1155/2012/958673, PMID: 22973521 PMC3438877

[48] Dai Q Song F Li X Huang F Zhao H. Comprehensive analysis of the expression and prognosis for IQ motif-containing GTPase-activating proteins in hepatocellular carcinoma. BMC Cancer. (2022) 22:1121. doi: 10.1186/s12885-022-10204-3, PMID: 36320006 PMC9628040

[49] Schmidt VA Chiariello CS Capilla E Miller F Bahou WF. Development of hepatocellular carcinoma in Iqgap2-deficient mice is IQGAP1 dependent. Mol Cell Biol. (2008) 28:1489–502. doi: 10.1128/MCB.01090-07, PMID: 18180285 PMC2258764

[50] Xia FD Wang ZL Chen HX Huang Y Li JD Wang ZM. Differential expression of IQGAP1/2 in Hepatocellular carcinoma and its relationship with clinical outcomes. Asian Pac J Cancer Prev. (2014) 15:4951–6. doi: 10.7314/APJCP.2014.15.12.4951, PMID: 24998570

[51] Jin X Liu Y Liu J Lu W Liang Z Zhang D. The overexpression of IQGAP1 and β-catenin is associated with tumor progression in hepatocellular carcinoma *in vitro* and *in vivo* . PloS One. (2015) 10:e0133770. doi: 10.1371/journal.pone.0133770, PMID: 26252773 PMC4529304

[52] Delgado ER Erickson HL Tao J Monga SP Duncan AW Anakk S. Scaffolding Protein IQGAP1 Is Dispensable, but Its Overexpression Promotes Hepatocellular Carcinoma via YAP1 Signaling. Mol Cell Biol. (2021) 41:1,7,10. doi: 10.1128/MCB.00596-20, PMID: 33526450 PMC8088129

[53] Zoheir KMA Abd-Rabou AA Darwish AM Abdelhafez MA Mahrous KF. Inhibition of induced-hepatic cancer *in vivo* through IQGAP1-shRNA gene therapy and modulation of TRAIL-induced apoptosis pathway. Front Oncol. (2022) 12:998247. doi: 10.3389/fonc.2022.998247, PMID: 36276098 PMC9581201

[54] He C Jaffar Ali D Qi Y Li Y Sun B Liu R. Engineered extracellular vesicles mediated CRISPR-induced deficiency of IQGAP1/FOXM1 reverses sorafenib resistance in HCC by suppressing cancer stem cells. J Nanobiotechnology. (2023) 21:154. doi: 10.1186/s12951-023-01902-6, PMID: 37202772 PMC10193671

[55] Llovet JM Kelley RK Villanueva A Singal AG Pikarsky E Roayaie S. Hepatocellular carcinoma. Nat Rev Dis Primers. (2021) 7:6. doi: 10.1038/s41572-020-00240-3, PMID: 33479224 PMC13537433

[56] Li S Wang Q Chakladar A Bronson RT Bernards A. Gastric hyperplasia in mice lacking the putative Cdc42 effector IQGAP1. Mol Cell Biol. (2000) 20:697–701. doi: 10.1128/MCB.20.2.697-701.2000, PMID: 10611248 PMC85173

[57] Gobbi PG Bergonzi M Comelli M Villano L Pozzoli D Vanoli A. The prognostic role of time to diagnosis and presenting symptoms in patients with pancreatic cancer. Cancer Epidemiol. (2013) 37:186–90. doi: 10.1016/j.canep.2012.12.002, PMID: 23369450

[58] Harada A Matsumoto S Yasumizu Y Shojima K Akama T Eguchi H. Localization of KRAS downstream target ARL4C to invasive pseudopods accelerates pancreatic cancer cell invasion. Elife. (2021) 10:12–14. doi: 10.7554/eLife.66721.sa2, PMID: 34590580 PMC8598236

[59] Li JH McMillan RH Begum A Gocke CB Matsui W. IQGAP1 maintains pancreatic ductal adenocarcinoma clonogenic growth and metastasis. Pancreas. (2019) 48:94–8. doi: 10.1097/MPA.0000000000001198, PMID: 30540680 PMC6293988

[60] Song C Wang G Liu M Han S Dong M Peng M. Deciphering the SOX4/MAPK1 regulatory axis: a phosphoproteomic insight into IQGAP1 phosphorylation and pancreatic Cancer progression. J Transl Med. (2024) 22:602. doi: 10.1186/s12967-024-05377-3, PMID: 38943117 PMC11212360

[61] Jin X Pan Y Wang L Ma T Zhang L Tang AH. Fructose-1,6-bisphosphatase inhibits ERK activation and bypasses gemcitabine resistance in pancreatic cancer by blocking IQGAP1-MAPK interaction. Cancer Res. (2017) 77:4328–41. doi: 10.1158/0008-5472.CAN-16-3143, PMID: 28720574 PMC5581962

[62] Hu W Wang Z Zhang S Lu X Wu J Yu K. IQGAP1 promotes pancreatic cancer progression and epithelial-mesenchymal transition (EMT) through Wnt/β-catenin signaling. Sci Rep. (2019) 9:7539. doi: 10.1038/s41598-019-44048-y, PMID: 31101875 PMC6525164

[63] Zhang B Cheng X Zhan S Jin X Liu T. MIB1 upregulates IQGAP1 and promotes pancreatic cancer progression by inducing ST7 degradation. Mol Oncol. (2021) 15:3062–75. doi: 10.1002/1878-0261.12955, PMID: 33793053 PMC8564634

[64] Razidlo GL Burton KM McNiven MA. Interleukin-6 promotes pancreatic cancer cell migration by rapidly activating the small GTPase CDC42. J Biol Chem. (2018) 293:11143–53. doi: 10.1074/jbc.RA118.003276, PMID: 29853638 PMC6052214

[65] Arnold M Park JY Camargo MC Lunet N Forman D Soerjomataram I. Is gastric cancer becoming a rare disease? A global assessment of predicted incidence trends to 2035. Gut. (2020) 69:823–9. doi: 10.1136/gutjnl-2019-320234, PMID: 32001553 PMC8520492

[66] Wu Y Tao Y Chen Y Xu W. RhoC regulates the proliferation of gastric cancer cells through interaction with IQGAP1. PloS One. (2012) 7:e48917. doi: 10.1371/journal.pone.0048917, PMID: 23145020 PMC3492142

[67] Papadaki V Erpapazoglou Z Kokkori M Rogalska ME Potiri M Birladeanu A. IQGAP1 mediates the communication between the nucleus and the mitochondria via NDUFS4 alternative splicing. NAR Cancer. (2023) 5:zcad046. doi: 10.1093/narcan/zcad046, PMID: 37636315 PMC10448856

[68] Ding H Liu Y Lu X Liu A Xu Q Yuan Y. Pepsinogen C interacts with IQGAP1 to inhibit the metastasis of gastric cancer cells by suppressing rho-GTPase pathway. Cancers (Basel). (2024) 16:5, 11. doi: 10.3390/cancers16101796, PMID: 38791874 PMC11120368

[69] Cai Q Zhu C Yuan Y Feng Q Feng Y Hao Y. Development and validation of a prediction rule for estimating gastric cancer risk in the Chinese high-risk population: a nationwide multicentre study. Gut. (2019) 68:1576–87. doi: 10.1136/gutjnl-2018-317556, PMID: 30926654 PMC6709770

[70] Li X Wang J. Mechanical tumor microenvironment and transduction: cytoskeleton mediates cancer cell invasion and metastasis. Int J Biol Sci. (2020) 16:2014–28. doi: 10.7150/ijbs.44943, PMID: 32549750 PMC7294938

[71] Gorisse L Li Z Wagner CD Worthylake DK Zappacosta F Hedman AC. Ubiquitination of the scaffold protein IQGAP1 diminishes its interaction with and activation of the Rho GTPase CDC42. J Biol Chem. (2020) 295:4822–35. doi: 10.1074/jbc.RA119.011491, PMID: 32094223 PMC7152761

[72] Xie W Han Z Zuo Z Xin D Chen H Huang J. ASAP1 activates the IQGAP1/CDC42 pathway to promote tumor progression and chemotherapy resistance in gastric cancer. Cell Death Dis. (2023) 14:124. doi: 10.1038/s41419-023-05648-9, PMID: 36792578 PMC9932153

[73] Yan J Yang Y Zhang H King C Kan HM Cai Y. Menin interacts with IQGAP1 to enhance intercellular adhesion of beta-cells. Oncogene. (2009) 28:973–82. doi: 10.1038/onc.2008.435, PMID: 19079338 PMC2645484

[74] Ren F Guo Q Zhou H. Menin represses the proliferation of gastric cancer cells by interacting with IQGAP1. BioMed Rep. (2023) 18:27. doi: 10.3892/br.2023.1609, PMID: 36909940 PMC9996331

[75] Davidson KW Barry MJ Mangione CM Cabana M Caughey AB Davis EM. Screening for colorectal cancer: US preventive services task force recommendation statement. Jama. (2021) 325:1965–77. doi: 10.1001/jama.2021.6238, PMID: 34003218

[76] Ding C Luo J Li L Li S Yang L Pan H. Gab2 facilitates epithelial-tomesenchymal transition via the MEK/ERK/MMP signaling in colorectal cancer. J Exp Clin Cancer Res. (2016) 35:5. doi: 10.1186/s13046-015-0280-0, PMID: 26754532 PMC4709914

[77] Hu HF Xu WW Li YJ He Y Zhang WX Liao L. Anti-allergic drug azelastine suppresses colon tumorigenesis by directly targeting ARF1 to inhibit IQGAP1-ERK-Drp1-mediated mitochondrial fission. Theranostics. (2021) Jan 1;11(4):1828-44. doi: 10.7150/thno.48698, PMID: 33408784 PMC7778598

[78] Zhang T Wang Z Liu Y Huo Y Liu H Xu C. Plastin 1 drives metastasis of colorectal cancer through the IQGAP1/Rac1/ERK pathway. Cancer Sci. (2020) 111:2861–71. doi: 10.1111/cas.v111.8, PMID: 32350953 PMC7419044

[79] Roma-Rodrigues C Mendes R Baptista PV Fernandes AR. Targeting tumor microenvironment for cancer therapy. Int J Mol Sci. (2019) 20:3–8. doi: 10.3390/ijms20040840, PMID: 30781344 PMC6413095

[80] Deng S Cheng D Wang J Gu J Xue Y Jiang Z. MYL9 expressed in cancer-associated fibroblasts regulate the immune microenvironment of colorectal cancer and promotes tumor progression in an autocrine manner. J Exp Clin Cancer Res. (2023) 42:294. doi: 10.1186/s13046-023-02863-2, PMID: 37926835 PMC10626665

[81] Huang YT Hsu YT Wu PY Yeh YM Lin PC Hsu KF. Tight junction protein cingulin variant is associated with cancer susceptibility by overexpressed IQGAP1 and Rac1-dependent epithelial-mesenchymal transition. J Exp Clin Cancer Res. (2024) 43:65. doi: 10.1186/s13046-024-02987-z, PMID: 38424547 PMC10905802

[82] Corcoran RB Ebi H Turke AB Coffee EM Nishino M Cogdill AP. EGFR-mediated re-activation of MAPK signaling contributes to insensitivity of BRAF mutant colorectal cancers to RAF inhibition with vemurafenib. Cancer Discov. (2012) 2:227–35. doi: 10.1158/2159-8290.CD-11-0341, PMID: 22448344 PMC3308191

[83] Zoheir K Mahmoud K Harisa GI Ashour A Abdel-Hamied HE Amara AA. Novel approach using shRNA of IQGAP1 for colon cancer therapy: HCT116 as a surrogate model colorectal carcinoma. Asian Pac J Cancer Prev. (2022) 23:2387–95. doi: 10.31557/APJCP.2022.23.7.2387, PMID: 35901346 PMC9727335

[84] Louis DN Perry A Wesseling P Brat DJ Cree IA Figarella-Branger D. The 2021 WHO classification of tumors of the central nervous system: a summary. Neuro Oncol. (2021) 23:1231–51. doi: 10.1093/neuonc/noab106, PMID: 34185076 PMC8328013

[85] Ghosh D Nandi S Bhattacharjee S. Combination therapy to checkmate Glioblastoma: clinical challenges and advances. Clin Transl Med. (2018) 7:33. doi: 10.1186/s40169-018-0211-8, PMID: 30327965 PMC6191404

[86] Han F Hu R Yang H Liu J Sui J Xiang X. PTEN gene mutations correlate to poor prognosis in glioma patients: a meta-analysis. Onco Targets Ther. (2016) 9:3485–92. doi: 10.2147/OTT.S99942, PMID: 27366085 PMC4913532

[87] Zhang F Lv M He Y. Identification of a novel disulfideptosis-related gene signature for prognostic implication in lower-grade gliomas. Aging (Albany NY). (2024) 16:6054–67. doi: 10.18632/aging.205688, PMID: 38546389 PMC11042955

[88] Cui X Song L Bai Y Wang Y Wang B Wang W. Elevated IQGAP1 and CDC42 levels correlate with tumor Malignancy of human glioma. Oncol Rep. (2017) 37:768–76. doi: 10.3892/or.2016.5341, PMID: 28035419 PMC5355752

[89] Zhang Q Zheng J Wu W Lian H Iranzad N Wang E. TRIM56 acts through the IQGAP1-CDC42 signaling axis to promote glioma cell migration and invasion. Cell Death Dis. (2023) 14:178. doi: 10.1038/s41419-023-05702-6, PMID: 36870986 PMC9985612

[90] Okura H Golbourn BJ Shahzad U Agnihotri S Sabha N Krieger JR. A role for activated Cdc42 in glioblastoma multiforme invasion. Oncotarget. (2016) 7:56958–75. doi: 10.18632/oncotarget.10925, PMID: 27486972 PMC5302965

[91] Lalatsa A Schatzlein AG Uchegbu IF. Strategies to deliver peptide drugs to the brain. Mol Pharm. (2014) 11:1081–93. doi: 10.1021/mp400680d, PMID: 24601686

[92] Lu SH Jiang XJ Xiao GL Liu DY Yuan XR. miR-124a restoration inhibits glioma cell proliferation and invasion by suppressing IQGAP1 and β-catenin. Oncol Rep. (2014) 32:2104–10. doi: 10.3892/or.2014.3455, PMID: 25175832

[93] Gao C Liang C Nie Z Liu Y Wang J Zhang D. Alkannin inhibits growth and invasion of glioma cells C6 through IQGAP/mTOR signal pathway. Int J Clin Exp Med. (2015) 8:5287–94., PMID: 26131103 PMC4483937

[94] An C Sun F Liu C Huang S Xu T Zhang C. IQGAP1 promotes mitochondrial damage and activation of the mtDNA sensor cGAS-STING pathway to induce endothelial cell pyroptosis leading to atherosclerosis. Int Immunopharmacol. (2023) 123:110795. doi: 10.1016/j.intimp.2023.110795, PMID: 37597406

[95] Hou L Hsu A Luo H Yuki K. IQGAP1 influences neutrophil maturation and its effector functions. Eur J Immunol. (2025) 55:e202451349. doi: 10.1002/eji.202451349, PMID: 39931750 PMC12418024

[96] Jeong HW Li Z Brown MD Sacks DB. IQGAP1 binds Rap1 and modulates its activity. J Biol Chem. (2007) 282:20752–62. doi: 10.1074/jbc.M700487200, PMID: 17517894

[97] Sullivan DP Dalal PJ Jaulin F Sacks DB Kreitzer G Muller WA. Endothelial IQGAP1 regulates leukocyte transmigration by directing the LBRC to the site of diapedesis. J Exp Med. (2019) 216:2582–601. doi: 10.1084/jem.20190008, PMID: 31395618 PMC6829592

[98] Wen X Li D Wang H Zhang D Song J Zhou Z. IQGAP1 domesticates macrophages to favor mycobacteria survival via modulating NF-κB signal and augmenting VEGF secretion. Int Immunopharmacol. (2024) 138:112549. doi: 10.1016/j.intimp.2024.112549, PMID: 38944950

[99] Hedman AC Li Z Gorisse L Parvathaneni S Morgan CJ Sacks DB. IQGAP1 binds AMPK and is required for maximum AMPK activation. J Biol Chem. (2021) 296:100075. doi: 10.1074/jbc.RA120.016193, PMID: 33191271 PMC7948462

[100] Dong H Liu R Zou K Jin Z Kang J Zhang Y. Higenamine promotes osteogenesis via IQGAP1/SMAD4 signaling pathway and prevents age- and estrogen-dependent bone loss in mice. J Bone Miner Res. (2023) 38:775–91. doi: 10.1002/jbmr.4800, PMID: 36907987

[101] van Reij RRI Hoofwijk DMN Rutten BPF Weinhold L Leber M Joosten EAJ. The association between genome-wide polymorphisms and chronic postoperative pain: a prospective observational study. Anaesthesia. (2020) 75 Suppl 1:e111–e20. doi: 10.1111/anae.14832, PMID: 31903573 PMC6973279

[102] Khan S Patra PH Somerfield H Benya-Aphikul H Upadhya M Zhang X. IQGAP1 promotes chronic pain by regulating the trafficking and sensitization of TRPA1 channels. Brain. (2023) 146:2595–611. doi: 10.1093/brain/awac462, PMID: 36477832 PMC10232262

[103] Shi J Chi Y Wang X Zhang Y Tian L Chen Y. MiR-124 regulates IQGAP1 and participates in the relationship between morphine dependence susceptibility and cognition. Front Psychiatry. (2022) 13:845357. doi: 10.3389/fpsyt.2022.845357, PMID: 35401251 PMC8983956

